# Evaluating Variability in Median Lingual Canal Morphology of the Anterior Mandible Using Cone-Beam Computed Tomography (CBCT) in the Melmaruvathur Population: A Retrospective Study

**DOI:** 10.7759/cureus.82978

**Published:** 2025-04-25

**Authors:** Sakshi Madhok, Kiruthika Sivakumar, D. Thirumal Rao, Soneya P., Sonia Abraham, Karthik V. C.

**Affiliations:** 1 Department of Prosthodontics, Crown and Bridge, Adhiparasakthi Dental College and Hospital, Melmaruvathur, IND; 2 Department of Prosthodontics, Crown and Bridge, Indira Gandhi Institute of Dental Science and Research, Pondicherry, IND

**Keywords:** cone-beam computed tomography, cross-section anterior mandible, interforaminal region, lateral vascular canal, median vascular canal

## Abstract

Objectives

The dimensions, shape, and number of lingual canals are critical for presurgical assessment of dental implants. The purpose of this cross-sectional study was to assess the frequency, distribution, position, and morphometric variations of the median lingual canal (MLC) in the Melmaruvathur population of South India.

Materials and methods

We conducted this study on a sample size of 200 patients of the Melmaruvathur region in Tamil Nadu with a male to female ratio of 1:1. We procured cone-beam computed tomography (CBCT) scans from the radiological archives of the Department of Implantology, Adhiparasakthi Dental College and Hospital, allowing us to study the interforaminal region. We identified MLCs in cross-sectional and axial slices and evaluated frequency, location, and parametric variations with respect to the inferior border of the mandible (IBM) and the buccal plate. We assessed dimensions of the canal, considering 1 mm as the critical diameter potentially leading to bleeding complications if encroached upon during surgical intervention.

Results

Lingual canals were visible in 192 (96%) patients. None was present in eight (4%) patients. In total, 336 MLCs were present. The majority of subjects (52%) had two canals; 57% of median vascular canals (MVC) were supraspinosum, and 41% of MLCs had a diameter of more than 1 mm. Gender association with canal diameter was nonsignificant.

Conclusion

Morphometric analysis showed significant variability in the pattern of presence, distribution, and frequency of MLCs in the population. Thorough preoperative clinical and radiographic assessment of the patient is crucial to prevent untoward intraoperative incidents, thus minimizing functional impairments and surgical morbidity

## Introduction

The mandible plays a pivotal role in the structural integrity and function of the craniofacial complex. The presence and characteristics of the median vascular canal (MVC) in the interforaminal region hold significant clinical and surgical relevance, particularly in the context of dental implantology and oral surgery. The MVC houses vascular structures, including the anastomosing vessels of the sublingual and submental arteries, and serves as a conduit for their passage through the mandible. Understanding the variations in the morphology and prevalence of the MVC is crucial for safe and successful dental implant placement and other surgical procedures in the anterior mandible, such as harvesting autogenous bone graft. This region, once considered safe, contains multiple critical structures that have been controversial for years and have reportedly led to life-threatening complications and hemorrhages [1-3]. The content of the lingual canal is also a matter of debate, as certain authors claim it to contain only vascular elements, whereas others believe it to have neurovascular content [4]. The most comprehensive study showed that the content of the lingual foramen is an artery resulting from the anastomosis of the sublingual branches of the right and left sublingual arteries [5]. Researchers have given several terminologies to these neurovascular bundles, such as median lingual canal (MLC), mandibular incisive canal, mandibular symphyseal canal, lingual vascular canal, and mandibular genial spinal canal [6]. Depending on the location, that is, if the canal is in the center of the mandible, it is known as MLC; if laterally positioned, it is known as the lateral lingual canal, which is considered an accessory [4]. The MLC is further divided into three types depending on the location with reference to genial tubercles in the midline: supraspinosum, intraspinosum, and infraspinosum [7].

Considering the criticality of the lingual vascular canal for implant surgery, in recent years, there has been growing interest in investigating the prevalence and morphology of the MVC, particularly in specific population groups, to better understand its anatomical variations and clinical implications. A systematic review and meta-analysis conducted in 2022 from 18 nationalities revealed a high prevalence (90.11%) of MLC, which differed significantly from region to region [8]. Similar studies were conducted earlier in the North Indian population and the Karnataka region of South India [9,10]. To fill the information lacuna for the population of Melmaruvathur in Tamil Nadu, South India, we conducted a cone-beam computed tomography (CBCT) study to identify the prevalence and morphometric variations in MLC in a sample of the Melmaruvathur population located in the Kanchipuram region of Tamil Nadu state in India. This population presents a unique demographic profile and genetic makeup, which may influence the characteristics of the MVC in this cohort. The insights gained from this study have the potential to enhance our understanding of mandibular anatomy and contribute to improved treatment outcomes in dental implantology and oral surgery.

Our aim in this study was to assess the presence, position, morphometric variations, and course of the MLC within the Tamil Nadu subpopulation. We also aimed to compare the data between men and women.

## Materials and methods

Study design

We conducted a cross-sectional retrospective study to investigate the prevalence and variation of the MVC and MLC in the anterior mandible of the Melmaruvathur population using CBCT. The Institutional Review Board of Adhiparasakthi Dental College and Hospital (APDCH), Melmaruvathur (ECR/1742/APDCH PROS-35/TN/2024), approved the study protocol, and we conducted all procedures in accordance with the principles outlined in the Declaration of Helsinki.

Participant recruitment and CBCT imaging protocol

We recruited 200 participants from the Melmaruvathur population for the study. As a standard institutional protocol, all patients signed consent forms before undergoing CBCT. Based on the median lingual foramen prevalence of 86.6%, we calculated the sample size as 179 using OpenEpi, version 3 (www.OpenEpi.com) [11]. We randomly procured CBCTs of the mandible from the archives of APDCH, Department of Radiology, spanning January 2022 to July 2024. We took no CBCT, especially for the study. Inclusion criteria comprised partially edentulous individuals aged 21 to 72 years with at least one mandibular anterior tooth missing and no history of craniofacial trauma, past surgery, or congenital anomalies. We excluded from the study unclear CBCTs, CBCTs without radiographic stents of partially edentulous mandibles, CBCTs with small and large field of view (FOV), history of orthodontic treatment, osteoporosis, and known cases of syndrome. We acquired all CBCTs using a CBCT machine (Dentsply Sirona), with the scanner operating at 85 kVp and 6 mA, medium FOV (8 x 8 cm) reconstructed, and read at a slice thickness of 1 mm.

Evaluation of canals

**Figure 1:**
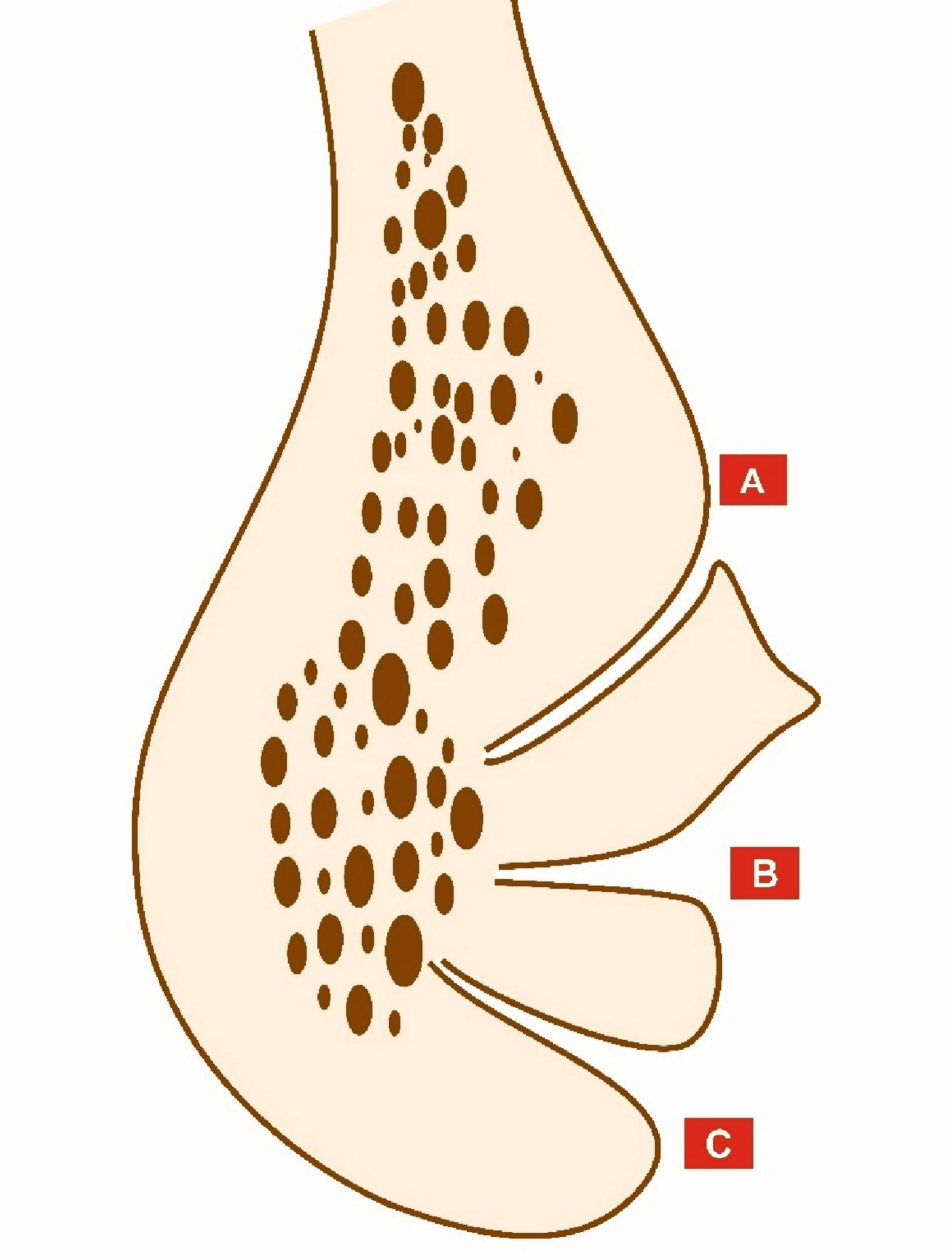
Diagramatic representation of the cross-section of MVC depicting location and course (A) Supraspinosum with caudal course. (B) Intraspinosum with transverse course. (C:) Infraspinosum with cephalic course. MVC: median vascular canal Figure created by the authors

Two observers independently evaluated CBCT images using dedicated CareStream 3D imaging software version 3.10.38 (Carestream Dental LLC, Atlanta, USA). To optimize image visualization, the observers used adjustable brightness and contrast. Independent examiners assessed all CBCTs and noted down values with consensus up to a single decimal point. Two implantologists, both with more than 10 years of experience in implantology, read the CBCTs. An oral radiologist reevaluated cases with disagreement, and we considered his findings final. We assessed the presence, location, and morphology of the MVC using axial and cross-sectional slices. To begin, we identified the mental foramen and cropped the image beyond it. We then identified the MVC and analyzed it for the number and position of MLC with respect to the genial tubercles, described as supraspinosum, intraspinosum, and infraspinosum, as well as its course, classified as caudal, transverse, and cephalic. Various morphometric parameters measured in cross-section included the diameter of the MVC at emergence, the diameter of the MVC at exit, the distance between MVC emergence and the buccal plate, the distance between MVC exit and the buccal plate, the distance between MVC emergence and the inferior border of the mandible (IBM), and the distance between MVC exit and the IBM (Figure 1).

Statistical analysis

We used descriptive statistics to summarize the characteristics of the study population and the prevalence of the MVC. We used IBM SPSS Statistics version 20 (IBM Corp., Armonk, USA). We analyzed the association between canal prevalence, position, demographic variables, and gender using the chi-squared test and the Mann-Whitney U test. Statistical significance was set at p < 0.05.

## Results

A total of 336 MVCs were present in 200 CBCT scans with a male-to-female ratio of 1:1 and a mean age of 44.4 ± 13.6 years. The mean ages of women and men were 42.2 ± 12.2 and 46.6 ± 14.6, respectively.

Median lingual foramen

The MLC was visible in 192 (96%) patients and not visible in eight (4%) patients. It was present in 92 scans (92%) of the female sample and in 100 scans (100%) of the male sample, with a statistically significant difference between the genders (Table 1).

**Table 1:** Number of MLCs MLC: median lingual canal

Gender	0, n (%)	1, n (%)	2, n (%)	3, n (%)	χ²	p-value
Male	0 (0%)	32 (16%)	56 (28%)	12 (6%)	9.61	0.022
Female	8 (4%)	36 (18%)	48 (24%)	8 (4%)		
Total	8 (4%)	68 (34%)	104 (52%)	20 (10%)		

With respect to MLC distribution, eight (4%) patients had no canal, 68 (34%) patients had a single canal, 104 (52%) had two canals, and 20 (10%) had three canals. None had four canals. Table 1 displays the distribution of canal numbers in men and women. Of the total 336 MVCs, 192 (57%) were supraspinosum, 76 (23%) were infraspinosum, and 68 (20%) were intraspinosum. Table 2 shows the association between MLC position and gender.

**Table 2:** Location of MLCs MLC: median lingual canal

Variables	Supraspinosum, n (%)	Infraspinosum, n (%)	Intraspinosum, n (%)	χ²	p-value
Male	100 (50%)	68 (34%)	12 (6%)	Supraspinosum: χ² = 8.20	0.004
Female	92 (46%)	8 (4%)	56 (28%)	Infraspinosum: χ² = 23.88	<0.001
				Intraspinosum: χ² = 23.08	<0.001

Of 336 MLCs, 199 (59%) had caudal course, 93 (28%) had cephalic course, and 44 (13%) had transverse course, with no association between the MLC course and gender. The distribution of MLCs by canal diameter revealed that 136 (41%) out of the total sample had canals with a critical diameter of the entry 1 mm or larger, with 60 (17.9%) women and 76 (22.6%) men. However, there was no statistically significant difference between the male and female samples. Two hundred (60%) canals had a noncritical diameter of less than 1 mm. Table 3 displays the values for linear measurements.

**Table 3:** Linear measurements in the male and female patients for MVCs MVC: median vascular canal; IBM: inferior border of the mandible

Morphometric Parameters	Variable	Total	Male	Female	t / χ²	p-value
Diameter of MVC emergence	Mean ± SD	0.910 ± 0.268	0.949 ± 0.279	0.867 ± 0.249	t = 2.46	0.015
	Median (Min–Max)	0.90 (0.40–1.60)	0.90 (0.40–1.60)	0.90 (0.40–1.40)		
Diameter of MVC exit	Mean ± SD	0.614 ± 0.225	0.613 ± 0.189	0.615 ± 0.260	t = -0.91	0.109
	Median (Min–Max)	0.60 (0.20–1.70)	0.60 (0.20–1.30)	0.60 (0.20–1.70)		
Distance from buccal plate (emergence)	Mean ± SD	10.36 ± 2.13	10.951 ± 2.022	9.605 ± 2.035	t = 4.11	<0.001
	Median (Min–Max)	10.25 (4.40–17.0)	10.60 (6.90–17.0)	9.60 (4.40–13.90)		
Distance from buccal plate (exit)	Mean ± SD	7.039 ± 1.726	7.284 ± 1.633	6.756 ± 1.790	t = 2.36	0.019
	Median (Min–Max)	7.15 (1.20–12.80)	7.30 (3.50–12.80)	6.80 (1.20–10.20)		
Distance from IBM (emergence)	Mean ± SD	10.414 ± 4.627	10.637 ± 4.625	10.156 ± 4.630	t = 1.29	0.198
	Median (Min–Max)	12.25 (0.30–18.20)	11.30 (2.30–18.20)	12.50 (0.30–16.10)		
Distance from IBM (exit)	Mean ± SD	9.633 ± 3.328	10.0 ± 3.216	9.210 ± 3.415	t = 1.94	0.054
	Median (Min–Max)	10.15 (2.60–16.0)	10.20 (2.70–16.0)	9.90 (2.60–15.80)		

## Discussion

The anterior mandible is particularly significant in orthognathic and implant surgery and as an autogenous bone donor site. Since 1986, reports have documented at least 19 life-threatening bleeding cases linked to dental implants in the interforaminal region [12]. An ultrasound study done on the lingual foramen showed the average diameter of the artery to be 1.41 ± 0.34 mm, with an average blood flow of 2.92 ± 3.19 mL/min, with higher values for men compared to women [13]. The amount of blood flow is a function of the radius of the cross-section of the artery (r²). In our study, we assessed the mean diameter of the canals. A visualization of lingual foramina larger than 1 mm is a red flag for the implant surgeon [14]. Based on this study, we considered the critical diameter of an artery that may cause profound hemorrhage to be 1 mm or more. We localized MLC as supraspinosum, intraspinosum, and infraspinosum [15] to provide insights into the potential implant length in the population. MLC should be considered when calculating the harvesting depth [16]. Therefore, we calculated the distance between the canal exit and the buccal plate to determine the bone harvesting depth for autogenous bone grafts. The pathway of lingual canals slopes as it transitions from lingual to buccal. Due to the inability to direct radiation perpendicular to the canal, 2D radiographs often misinterpret the radiopaque rim [5]. Thus, in our study, we measured the frequency of the buccolingual course of the canal, depicting it as caudal, cephalic, and transverse. This information provided data on the likelihood of missing the canal in a 2D radiograph.

Depending on the modality used to visualize MVC, considerable variation is visible in the visual prevalence of MVC. It is visible only in 28% of patients by periapical film technique, in 85% of patients by dry skull radiography, and in 96% of patients by reading CBCT [16]. Historically, researchers have studied mandibular vascular structures and variations in their morphometrics using cadavers [17], dry mandibles [18], ultrasound [13], 2D radiographs [19], multislice CT [20], and CBCT. We utilized CBCT to study the lingual canal because it is considered the most reliable and appropriate technique for diagnosing the presence of neurovascular canals [21,22].

Our study showed the prevalence of MLC in 96% of the tested population, with a statistically significant difference between men (100%) and women (92%). Kumar et al. showed a prevalence of 100% in the South Indian population [10]. Regarding the number of canals, we found that 52% of the subjects had two canals, while 34% had a single canal and 10% had three canals. No patient had four canals. The findings aligned with those of the Egyptian population and Rosano et al. [23], who documented that the majority of cases had two canals, followed by single canals, three canals, and no canals. In contrast to CBCTs scanned by Kumar et al. [10], the findings were as follows: single canal - 39%, double - 60%, three canals - 1%. Both percentage theorists utilized a sample size of 100 individuals.

In our study, the majority (57%) of MVCs were supraspinosum, 23% were infraspinosum, and 20% were intraspinosum. The results of a study in the Egyptian population [22] also align with these values, with the supraspinosum region having the highest percentage of canals, followed by the infraspinosum. In contrast, Kumar et al. [10] found that the majority (45%) of canals were infraspinosum, 39% were supraspinosum, and 16% were intraspinosum.

In our study, canals with a diameter of 1 mm or more were 46%, with no significant differences observed between men and women. In Moshfeghi et al.’s study on the Iranian population [11], 19% of MVCs crossed 1 mm in diameter. Kung et al. [16] reported 37.2% canals with a diameter of more than 1 mm in the Taiwanese population. They also observed a significant difference between male and female individuals, which contrasts with the findings of the current study. Kumar et al. [10] reported that 61% of MLCs crossed a diameter of 1 mm. The aforementioned differences can be attributed to the differences in geographical backgrounds of the samples and differences in sample size. It is worth mentioning that to keep a safety margin, we considered canals with diameters of 1 mm or more as critical, whereas the majority of the studies considered canals with diameters of more than one as critical. The mean diameter of canals in our study was 0.910 (± 0.268) mm, with a statistically significant difference between men and women. Mean diameters of MLC reported in Indian, Egyptian, Taiwanese, and Japanese populations were 1.07 (±0.44) mm [10], 1.68 (±1.27) mm [22], 1.20 (±0.25) mm [14], and 1.05 (±0.59) mm [24], respectively, in various studies. Again, this variation can be attributed to ethnic and geographical differences as well as the sample size used. Moro et al. [24] took 58 subjects from the Japanese population for their study. If surgery damages the MLC, a 2 mm diameter artery with an estimated blood flow of 0.2 mL/min can lose 420 mL of blood in 30 minutes [25]. Only 13% of canals in our study had a transverse linguobuccal trajectory; the rest all had a caudal or cephalic course. Other researchers also observed similar results [10,18]. This suggests that in the population we studied, the 2D imaging modality may miss 87% of canals.

Knowledge about the distance between the exit of MVC and the buccal cortical plate is crucial to negate any risk of bleeding during chin autograft interventional surgeries. Some experts recommend harvesting from the buccal plate in the anterior mandible at a depth of 4 mm [16,26]. In our study, we found that the mean distance between the buccal plate and the terminal end of MLC was 7.039 (±1.726) mm, with a statistically significant difference between men and women. In their study of people from Karnataka, South India, Kumar et al. also found a significant difference in the mean distance between the buccal plate and the exit of MLC between men (6.50 ± 4 mm) and women (6.00 ± 2.45 mm). This suggests that within the South Indian population, women are more likely to have complications associated with bleeding during chin bone graft retrieval due to a shorter mean distance. However, limited studies have been conducted to validate this finding. This value was found to be 5.44 ± 1.36 mm in the Taiwanese population, with a statistically significant difference between male and female individuals [16]. Elmasry et al. [22], with similar results (95.6 ± 1.9), found no statistical difference between male and female patients in their study on the Egyptian population.

It is important to measure bone thickness between the MLC and the inferior border of the mandible to exclude the possibility of hemorrhage during orthognathic surgeries [23]. We recorded the distance between the entry MVC and the inferior border of the mandible to be 10.41 (± 4.62) mm in our study. In close approximation to our study, Kumar et al. [10] found the distance to be 9.95 (±3.84) mm in men and 9.45 (±2.46) mm in women. Various other authors have also shown results that agree with ours: Wang et al. [14] reported a distance of 11.50 (±4.33) mm; Elmasry et al. [22] reported 11.42 (±5.28) mm; Rosano et al. [23] reported 12.2 (±3.0) mm; and Kung et al. [16] reported 12.68 (±3.02) mm, which were slightly larger than our results. None of the studies, including ours, showed an association with gender.

These variations can be attributed to various factors such as ethnic differences in the study population, sample size, study methodology, the CBCT machine used, CBCT settings in the machine, and slice thickness.

Limitations

Our study on the Tamil Nadu subpopulation has some limitations. The limited sample size implies that future research should encompass a broader population for more precise outcomes. We did not measure the distance of the canal from the alveolar crest in our study because our sample included partially edentulous mandibles with varying degrees of resorption, potentially introducing bias into the data. We did not evaluate the prevalence, distribution, or dimension of the lateral lingual canal. Additionally, we did not conduct a parametric association with the edentulous state of the patient and age [27,28]. Future researchers should investigate all these factors to determine any age-related or change-related to edentulousness in mandibular anatomy. Furthermore, to obtain a more representative sample, it would be beneficial to include participants from different demographic regions across Tamil Nadu, India.

## Conclusions

This CBCT study reveals a high prevalence of the MVC in the South Indian subpopulation, with gender being a significant contributing factor only in certain parameters. These findings have implications for dental implant placement, autogenous bone graft retrieval, and other surgical procedures within this population. The clinical implications of MVC variations and their impact on treatment outcomes warrant further investigation. These results highlight the importance of using CBCT scans prior to oral surgery and when planning dental implants.
